# Determinação do Tecido Cicatricial do Miocárdio no Fenômeno de Fluxo Coronário Lento e a Relação entre a Quantidade de Tecido Cicatricial e o Nt-ProBNP

**DOI:** 10.36660/abc.2018149

**Published:** 2020-03-20

**Authors:** Mustafa Candemir, Asife Şahinarslan, Merve Yazol, Yusuf Ali Öner, Bülent Boyacı

**Affiliations:** 1 Yozgat City HospitalDepartment of CardiologyYozgatTurquia Yozgat City Hospital - Department of Cardiology , Yozgat - Turquia; 2 Gazi UniversityFaculty of MedicineDepartment of CardiologyAnkaraTurquia Gazi University - Faculty of Medicine - Department of Cardiology , Ankara - Turquia; 3 Şanlıurfa Education and Research HospitalDepartment of RadiologyŞanlıurfaTurquia Şanlıurfa Education and Research Hospital , Department of Radiology , Şanlıurfa - Turquia; 4 Gazi UniversityFaculty of MedicineDepartment of RadiologyAnkaraTurquia Gazi University - Faculty of Medicine - Department of Radiology , Ankara - Turquia

**Keywords:** Insuficiência Cardiaca, Reserva Fracionada de Fluxo Miocárdico, Cicatriz Hipertrófica, Prognóstico, Peptídeo Natriurético Tipo C, Fibrose Endomiocárdica, Espectroscopia de Ressonância Magnética/métodos

## Abstract

**Fundamento:**

A fisiopatologia e o prognóstico não estão claramente determinados nos pacientes com fenômeno do fluxo coronário lento (FCL). Esses pacientes apresentam várias condições clínicas, que variam desde quadro assintomático até internação hospitalar com morte cardíaca súbita.

**Objetivos:**

Nosso objetivo foi avaliar os achados da ressonância magnética cardíaca (RMC) com o realce tardio pelo gadolínio (RTG), como um indicador de fibrose miocárdica. Também buscamos determinar a relação entre a presença de fibrose miocárdica e os níveis de NT-proBNP em pacientes com FCL na artéria coronária descendente anterior esquerda (DAE).

**Métodos:**

Ao todo, 35 pacientes, entre 31 e 75 anos de idade, foram incluídos. Os pacientes estudados (n=19) apresentaram artérias coronárias epicárdicas normais na angiografia, mas tinham FCL na DAE. O grupo controle de pacientes (n=16) apresentou artérias coronárias epicárdicas normais e níveis de escore TIMI normais na angiografia. Em ambos os grupos, os pacientes foram examinados com RMC para a detecção de presença de fibrose miocárdica. Além disso, níveis plasmáticos de NT-proBNP foram medidos. Valores de p < 0,05 foram considerados significativos.

**Resultados:**

A taxa de fibrose miocárdica foi significativamente maior na RMC para os pacientes com FCL (p=0.018). Uma quantidade variável de tecido cicatricial foi detectada no ápice ventricular esquerdo em 7 pacientes e nas regiões inferior e inferolateral em 3 pacientes. Não foram observadas diferenças nos níveis de NT-proBNP nos pacientes com FCL. Entretanto, os níveis de NT-proBNP foram maiores nos pacientes com FCL, que apresentaram fibrose miocárdica na RMC (p=0.022).

**Conclusões:**

Em suma, o RTG na RMC mostrou que a cicatriz miocárdica isquêmica pode estar presente nos pacientes com FCL. Esses resultados indicam que o FCL pode nem sempre ser inofensivo. (Arq Bras Cardiol. 2020; 114(3):540-551)

## Introdução

Há pouca informação na literatura em relação ao prognóstico do fenômeno do fluxo coronário lento (FCL). Dados preexistentes indicam que a isquemia miocárdica relacionada ao fluxo lento pode causar angina e o prognóstico é pior nesses pacientes. ^1^ Também relatou-se uma associação entre infarto agudo do miocárdio, ^2^ morte cardíaca súbita e arritmia ventricular maligna e o FCL. ^3^ A ocorrência de episódios recorrentes de dor torácica ou dor torácica em repouso, bem como elevadas taxas de internações de emergência e hospitalizações, foram relatadas. ^4 , 5^ Desse modo, esse fenômeno não é tão inofensivo como parece mas, ao contrário, possui potencial para causar séria deterioração na qualidade de vida. Não se sabe ao certo atualmente se há lesões orgânicas nesses pacientes, devido à ausência de investigações e resultados mais aprofundados.

Os níveis de N-terminal do pró-hormônio do peptídeo natriurético do tipo B (NT-proBNP) aumentaram após o exercício nos pacientes com fluxo coronário lento. ^6^ Há uma correlação entre a isquemia ou o tamanho do infarto na ressonância magnética (RMI), e essa correlação também pode ser observada nos níveis de NT-proBNP nos pacientes com síndrome coronariana aguda. ^7^ Com os avanços na ressonância magnética cardíaca (RMC), a isquemia microvascular e a fibrose cardíaca podem ser demonstradas através dessa técnica. ^8 , 9^ A relação entre a extensão da fibrose e os níveis de NT-proBNP nesses pacientes já foi revelada em vários exames de RMI realizados em pacientes com síndrome aguda coronariana. ^10 , 11^ Entretanto, não há estudos na literatura que tenham avaliado pacientes com FCL para detecção da presença de fibrose no tecido do miocárdio, de acordo com os resultados do realce tardio pelo gadolínio (RTG) na RMC. O objetivo deste estudo foi investigar a presença de fibrose miocárdica em pacientes com fluxo lento na artéria coronária descendente anterior esquerda por meio da técnica do realce tardio pelo gadolínio na RMC, bem como avaliar a relação entre a fibrose miocárdica e os níveis de NT-proBNP.

## Materiais e Métodos

### População do Estudo

Dentre os pacientes que foram admitidos no nosso departamento entre janeiro de 2015 e agosto de 2016, e submetidos à angiografia coronária devido a dores torácicas, 19 pacientes com o fenômeno do fluxo coronário lento na DAE foram incluídos neste estudo de coorte prospectivo. O grupo controle incluiu dezesseis pacientes cujas artérias epicárdicas estavam completamente normais com fluxo coronário normal.

Este estudo foi aprovado pelo Comitê de Ética do Hospital Universitário de Gazi e conduzido em conformidade com os princípios da declaração de Helsinki.

### Critérios de Exclusão

Os seguintes pacientes foram excluídos do estudo: pacientes com ectasia de artéria coronariana ou lesões ateroscleróticas nas artérias coronárias esquerda e descendente anterior esquerda; pacientes submetidos à intervenção coronária percutânea; pacientes com intervenção da artéria coronária agendada; pacientes com estenose >50% em qualquer artéria coronária; pacientes com história prévia de IM; pacientes com função sistólica do ventrículo esquerdo <50%; pacientes com claustrofobia, insuficiência cardíaca ou disfunção valvar, extra-sístoles ventriculares ou anormalidades da condução atrioventricular e bloqueio de ramo ou fibrilação atrial; pacientes com teste de esforço positivo; pacientes com cardiomiopatias restritiva, hipertrófica ou dilatada; pacientes com doença sistêmica conhecida (hipertireoidismo, hipotireoidismo, malignidade, doença autoimune, infecção ou quaisquer distúrbios pulmonares, hepáticos, renais, hematológicos); pacientes com história prévia de miocardite, com TFG <80 ml/min, e pacientes que se recusaram a participar do estudo.

### Dados dos Pacientes

Foram medidos a altura, o peso e o índice de massa corporal (IMC) dos pacientes do estudo. A idade, o gênero, os fatores de risco cardiovascular (hipertensão, diabetes, dislipidemia, tabagismo e histórico familiar), características demográficas e comorbidades foram registrados. Foi obtido eletrocardiograma (ECG) de todos os pacientes e todos eles apresentaram ritmo sinusal. Todos os pacientes do estudo foram examinados na posição decúbito lateral direita, utilizando um sistema de ultrassom Vivid 7-Pro (GE Vingmed, Horten, Noruega), equipado com um transdutor de 2,5 MHz, através do registro simultâneo pelo ECG de 1 derivação. Medições em modo-M e Doppler foram realizadas em conformidade com as recomendações da Associação Americana de Ecocardiografia. ^12^

O teste de esforço foi realizado em média 3 dias antes da angiografia em todos os pacientes (GE medical system, Milwaukee, EUA), de acordo com o protocolo padrão de Bruce, sendo o ECG convencional, além das medições da pressão arterial e da frequência cardíaca realizadas em períodos de tempo pré-definidos, conforme dizetrizes relevantes. ^13^

Foram coletadas amostras de sangue para dosagem de NT-proBNP, através da bainha vascular, imediatamente antes da sua retirada. Após a coleta, elas foram centrifugadas por 10 minutos a 4500 RPM e armazenadas a -20 ºC até a realização dos testes sorológicos. No dia da análise, após as amostras atingirem a temperatura ambiente, um imunoensaio eletroquimioluminescente foi realizado com o Analisador Cobas e 411 (Roche Diagnostics GmbH, Mannheim, Alemanha). Os resultados foram apresentados em picogramas por ml (pg/ml). O coeficiente de variação do NT-proBNP, através desse método, foi inferior a 5%.

### Angiografia Coronária e Contagem de Quadros TIMI

A angiografia coronária foi realizada utilizando-se a técnica padrão de Judkins com acesso femoral e a 30 imagens por segundo, através do sistema de angiografia Infinix (Toshiba Corporation, Tochigi, Japão). A iopromida (Ultravist-370; Bayer Pharma AG, Berlim, Alemanha) foi utilizada como agente de contraste durante a angiografia coronária. Uma média de 6 a 8 ml do agente de contraste foi injetada manualmente para cada exposição. As artérias coronárias foram visualizadas em projeção oblíqua esquerda e direita no ângulo cranial-caudal apropriado. A velocidade do fluxo na DAE foi avaliada nas projeções oblíquas anterior direita e esquerda, frequentemente com ângulo caudal. As imagens foram avaliadas por dois especialistas clínicos cegos em relação às condições clínicas dos pacientes.

A avaliação quantitativa do fluxo coronário foi realizada de acordo com o estudo TIMI-4, através da contagem de quadros angiográficos, a partir do momento da administração do agente de contraste, até a sua chegada a um determinado ponto distal. ^14^ A metodologia da contagem de quadros foi padronizada para cada vaso epicárdico. A contagem de quadros TIMI começou com o primeiro quadro no qual o contraste preencheu completamente a artéria. O preenchimento completo da artéria foi determinado pelo cumprimento dos três critérios a seguir: (1) Uma coluna de contraste total ou quase totalmente concentrado deve se estender ao longo de toda a origem da artéria coronária; (2) O contraste toca ambas as bordas de origem da artéria coronária; e (3) Deve haver um um fluxo anterógrado de contraste. O último quadro a ser contado foi aquele no qual o contraste preencheu inicialmente o ramo distal da artéria alvo. A opacificação completa do segmento distal não foi necessária.

O escore TIMI para a DAE foi 1,7 vez mais elevado do que a contagem média da ACD e da ACx. Consequentemente, a contagem do escore TIMI para a DAE foi dividida por 1,7 para calcular a contagem quadro a quadro TIMI corrigida (CTFC).

No nosso estudo, o fluxo coronário para a DAE foi considerado normal se a contagem do escore TIMI <23 e foi definida como lenta se a contagem do escore TIMI ≥ 23. ^15 , 16^

### Técnica da Ressonância Magnética

A RMC foi realizada após uma mediana de 8 dias (0-21 dias) após a angiografia coronária. Sequências padronizadas de estudos sobre perfusão pela RMC foram utilizadas em todos os pacientes. A veia antecubital esquerda foi usada para injeção do contraste intravenoso. Os dados de RM dos pacientes foram adquiridos utilizando um sistema de IRM 3T (Siemens MAGNETOM® Verio, Erlangen, Alemanha) equipado com gradiente de potência de 45 mT/m. Uma bobina de 6 canais foi colocada na parede torácica frontal enquanto o paciente estava deitado em decúbito dorsal com as almofadas de ECG posicionadas corretamente. Foram obtidas aquisições multiplanares com uma sequência turbo-FLASH de inversão-recuperação sensível à fase (PSIR) em múltiplas apneias expiratórias. As imagens padrão do coração de eixo longo, 2 câmaras, 4 câmaras e eixo curto foram obtidas alinhando a válvula mitral e o ápice. Os parâmetros de imagem foram: TR (tempo de repetição): 800; TE (tempo de eco) 6,66 ms; espessura das fatias: 8 mm; matriz: 128x256; FOV (campo de visão): 400 mm. Imagens ponderadas em T1 e T2 acompanhadas de sequência de pulsos “inversão-recuperação” para atenuação de sinais de sangue (sangue escuro) e de sequência-SPIN ECO foram adquiridas para avaliar a morfologia do miocárdio (TR/TE/ espessura/ matriz/ FOV: 698/6,6/ 8 mm/ 128x256, 360 mm). Foram adquiridas imagens de perfusão miocárdica dinâmica de primeira passagem uilizando-se a sequência de pulso SR Turbo FLASH (TfI), após administração de uma dose de 0,025 mmol/kg de Gd-DTPA intravenoso (Magnevist; Bayer Healthcare, Wayne NJ, EUA). Em intervalos de oito minutos de repouso, uma série de imagens de “gradiente-eco” (cine-RM) no plano de eixo curto foram adquiridas para avaliação funcional dos ventrículos ao longo do ciclo cardíaco, utilizando-se a apneia (TR/TE/ espessura/ matriz/ FOV: 40.24/ TE/ 8 mm/128x256/ 360 mm). Imagens ponderadas em T1, de eixo curto e 4 câmaras, foram obtidas na sequência PSIR, aproximadamente 8 minutos após a administração do contraste (TR/TE/ espessura/ matriz/ FOV: 756/ TE: 1,15/ 6 mm/ 128x256/360 mm). O tempo total de duração do exame foi de 35 minutos em média.

Os custos da RMC e da dosagem plasmática do NT-proBNP foram cobertos pela Unidade de Projetos de Pesquisa Científica da Universidade de Gazi.

### Análise de Imagem por Ressonância Magnética

Todas as imagens da RMC foram transferidas para a estação de trabalho para análise (Siemens, Leonardo multimodality workplace, Siemens Healthcare). Todas as avaliações foram realizadas visualmente. Os exames de RMC foram avaliados retrospectivamente por um radiologista com mais de 15 anos de experiência em imagem cardíaca, de forma cega aos resultados da ecocardiografia e da angiografia coronária. Ao se observar quaisquer defeitos de perfusão ou realce tardio durante essas avaliações, os mesmos eram registrados de forma precisa. O realce do contraste nas sequências de perfusão foi definido como o cumprimento de todas as 5 fases após a obtenção da intensidade de sinal mais alta no ventrículo esquerdo. Os resultados da RMC foram então comparados com os resultados da ecocardiografia e da angiografia coronária dos pacientes.

### Análise Estatística

Utilizou-se o programa estatístico SPSS (versão 21.0, SPSS Inc., Chicago, Illinois, EUA) para a análise de dados do estudo. Foram utilizados métodos visuais (histogramas, gráficos de probabilidade) e analíticos (teste Kolmogorov-Smirnov) para determinar a normalidade das variáveis. Nas análises descritivas, as variáveis distribuídas normalmente foram apresentadas como valor e desvio padrão, enquanto que as não normais foram apresentadas como mediana (intervalo interquartil). As variáveis categóricas foram apresentadas como percentuais. Amostras independentes do Teste-t e Mann-Whitney U foram usadas para comparar as variáveis numéricas. A análise do qui-quadrado de Pearson foi utilizada para comparar os dados categóricos, mas o teste exato de Fisher foi realizado quando dois dos valores esperados ficavam abaixo de 5 ou um dos valores esperados fosse menor que 2. Valores de p <0,05 foram considerados estatisticamente significativos.

## Resultados

**Figura 1:**
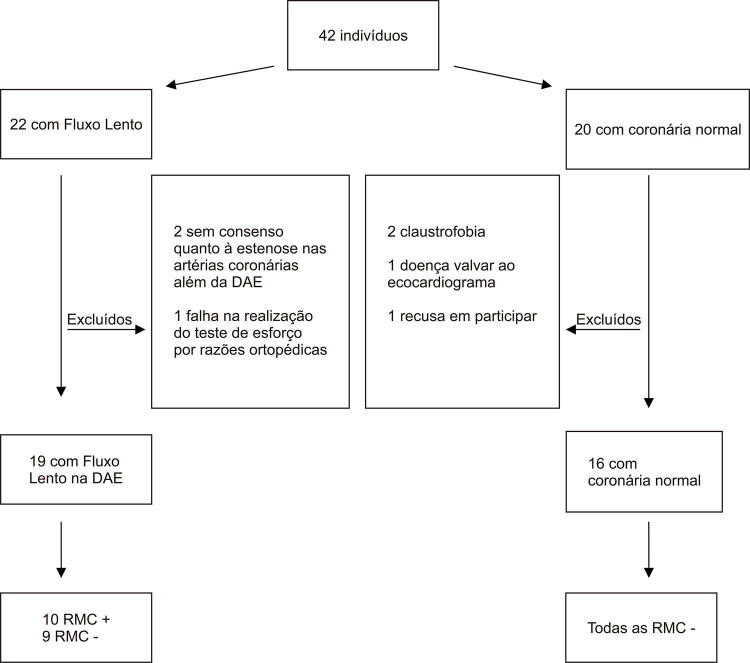
– *Seleção de pacientes. RMC: imagem por ressonância magnética cardíaca.*

Ao todo, 35 pacientes foram incluídos no estudo. Os pacientes foram divididos em 2 grupos: o grupo de pacientes e o grupo controle. Dezenove pacientes foram incluídos no grupo com fluxo lento na DAE, e 16 pacientes com fluxo coronário normal foram incluídos no grupo controle ( Figura 1 ). Os pacientes do grupo controle foram comparados, quanto aos fatores de risco, aos indivíduos dos grupos de pacientes. A idade média dos pacientes era de 50,3 ± 10,7, e 6 dos 35 pacientes (17%) eram mulheres. Onze pacientes (31%) eram diabéticos, 9 pacientes (25%) eram hipertensos, 5 pacientes (14%) eram dislipidêmicos, 18 pacientes (51%) eram tabagistas e 8 pacientes (22%) tinham histórico familiar positivo ( Tabela 1 ).

**Tabela 1:** – Comparação das características clínicas de ambos os grupos

Parâmetros	Total (N=35)	Fluxo Lento (N= 19)	Controle (N= 16)	Valor de p
Idade, média (DP), anos	50,3 ± 10,7	51,3 ± 8,2	49,44 ± 12,8	0,62
Sexo (Masculino), n (%)	29 (82)	15 (78,9)	14 (87,5)	0,50
Hipertensão, n (%)	9 (25)	6 (31,6)	3 (18,8)	0,38
Diabetes mellitus, n (%)	11 (31)	6 (31,6)	5 (31,3)	0,98
Tabagista, n (%)	18 (51)	9 (47,4)	9 (56,3)	0,60
Histórico familiar, n (%)	8 (22)	4 (21,1)	4 (25)	0,78
Dislipidemia, n (%)	5 (14)	3 (15,8)	2 (12,5)	0,78
IMC, média (DP) (kg/m ^2^ )	27,7 ± 2,3	28,1 ± 2,5	27,3 ± 2	0,39
NT-proBNP (pg/ml)	29,5 (17,7-66,2)	47,8 (22,6-121,5)	26,0 (10,9-58,1)	0,246
cTFC (quadro/segundo)	34,6 ± 16,2	28,0 ± 8,6	13,1 ± 1,2	<0,001
METs, mL/kg/dk	10,38 ± 1,91	9,74 ± 2,05	11,15 ± 1,43	0,027
Resultados positivos da IMR n (%)	10 (28)	10 (52,6)	0 (0)	0,001

*IMC: índice de massa corporal; cTFC: TIMI corrigido; METs: equivalentes metabólicos; RMI: ressonância magnética por imagem.*

A principal queixa de todos os pacientes era dor torácica. O ECG de todos os pacientes mostrou ritmo sinusal. A frequência cardíaca variou entre 64 e 92 bpm. A frequência cardíaca média foi 74 bpm. Além disso, não houve sinal de isquemia, hipertrofia ou arritmia ao ECG. A fração de ejeção do ventrículo esquerdo e outros achados ecocardiográficos dos pacientes estavam normais. Adicionalmente, os testes de esforço de todos os pacientes foram negativos. A troponina de alta sensibilidade foi medida antes e depois da angiografia coronária em todos os pacientes. Todos os valores ficaram abaixo do valor limite e não houve aumento nos valores de troponina após a angiografia.

O tempo de intervalo entre os exames de RMC e a angiografia coronária dos pacientes não deveria ser superior a 21 dias.

**Figura 2:**
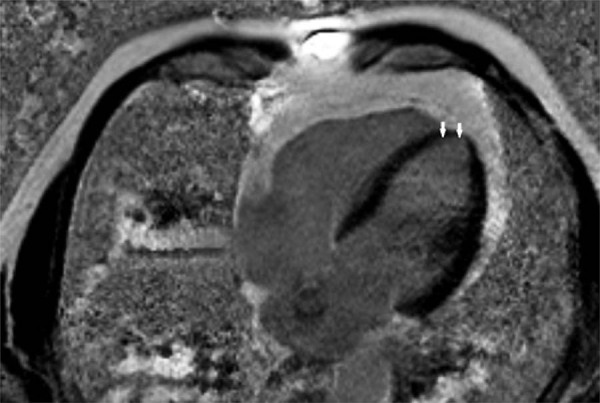
– *Imagem no plano 4 câmaras, na sequência inversão-recuperação sensível à fase (PSIR) com realce tardio, revelando realce tardio subendocárdico circunferencial na região apical (setas) do ventrículo esquerdo.*

**Figura 3:**
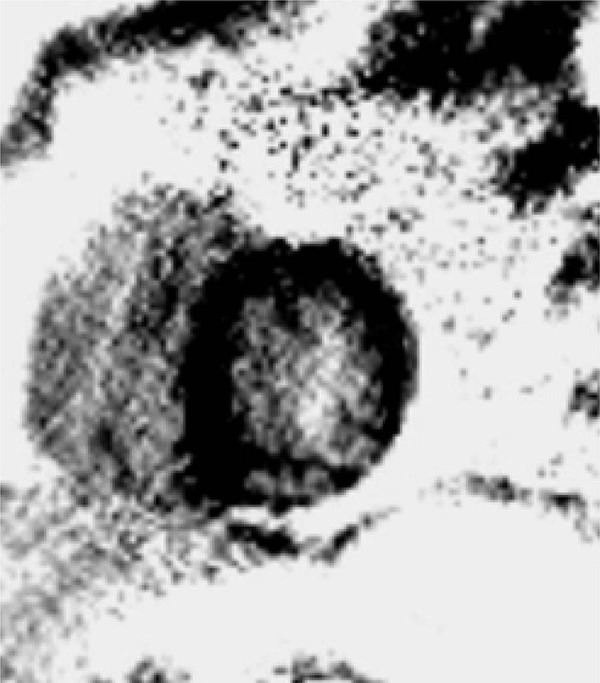
– *Imagem de RMC na sequência PSIR com contraste tardio no eixo curto, revelando áreas e focos de realce tardio em regiões subendocárdicas (transmurais) e subepicárdicas, sobretudo nas paredes do ventrículo esquerdo inferior e inferolateral.*

**Figura 4:**
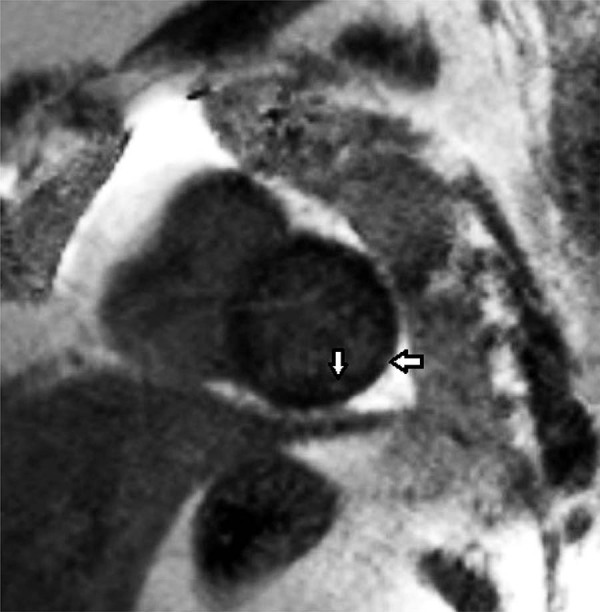
– *Imagem de RMC na sequência PSIR com contraste tardio no eixo curto, revelando focos de realce tardio em regiões subendocárdicas e subepicárdicas localizadas nas paredes inferior e inferolateral do VE (setas). Essas áreas hiperintensas refletem tecidos cicatriciais fibróticos que se espalham para o território da DAE.*

Quando os pacientes com fluxo lento foram comparados com o grupo controle, nenhuma diferença significativa foi observada nos valores de NT-proBNP (p=0,247). Os resultados positivos da RMC foram significativamente mais comuns nos pacientes com fluxo lento (p=0,001) (Table 1). Tecido cicatricial foi observado em níveis variáveis no ápice cardíaco de 7 pacientes ( Figuras 2 e 5 ) e nas regiões inferior e inferolateral em 3 pacientes ( Figures 3 , 4 , 6 , 7 and 8 ). Nenhum tecido cicatricial foi observado em 9 pacientes ( Figure 9 ).

**Figura 5:**
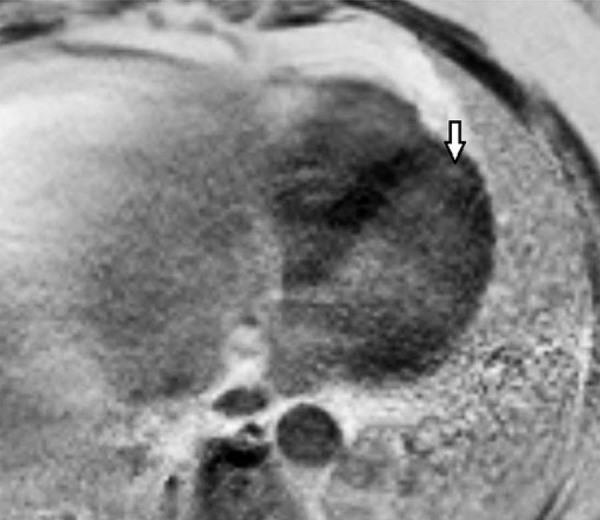
– *Lesão focal em imagem ponderada em T1 no plano 4 câmaras, na sequência PSIR, com realce tardio pelo gadolínio. Lesão focal localizada na região apical do ventrículo esquerdo, revelando realce tardio subendocárdico-miocárdico compatível com fibrose.*

**Figura 6:**
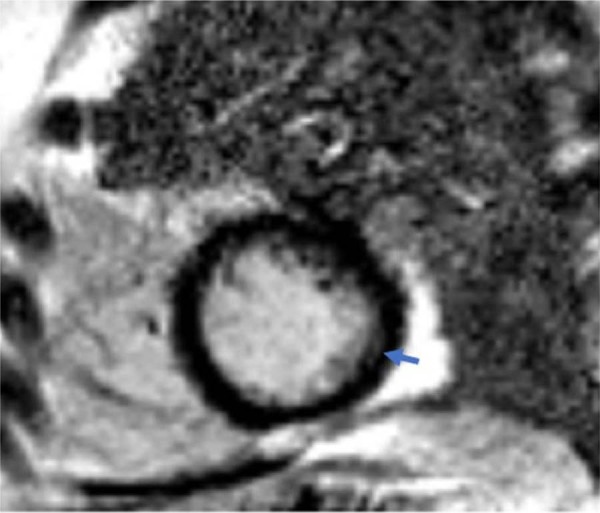
– *Imagem de sequência PSIR, com realce tardio, no eixo curto, representando áreas de realce subendocárdicas, sobretudo na parede ventricular esquerda inferolateral. Tecido cicatrial abrangendo quase 25-50 % da espessura da parede.*

**Figura 7:**
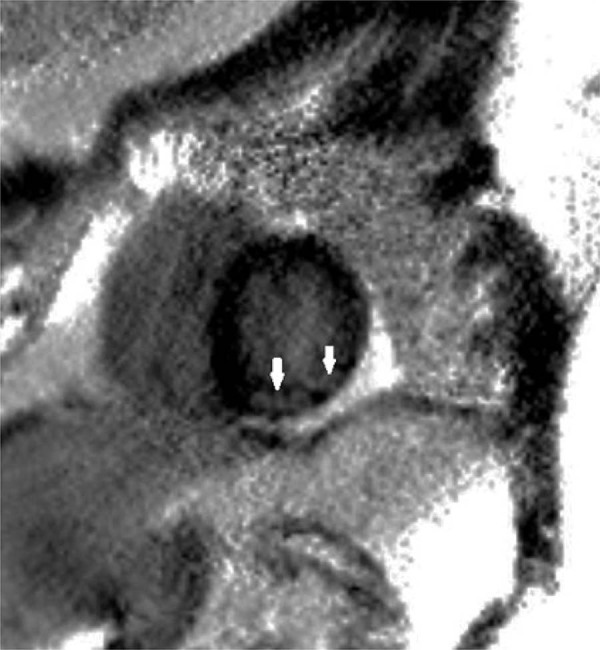
– *Imagem de RMC com sequência PSIR pela técnica do contraste tardio no eixo curto, revelando áreas de realce focal nas regiões subepicárdica (transmural) e subendocárdica, sobretudo nas paredes inferior e inferolateral do VE (setas), indicando tecidos cicatriciais na distribuição da DAE.*

**Figura 8:**
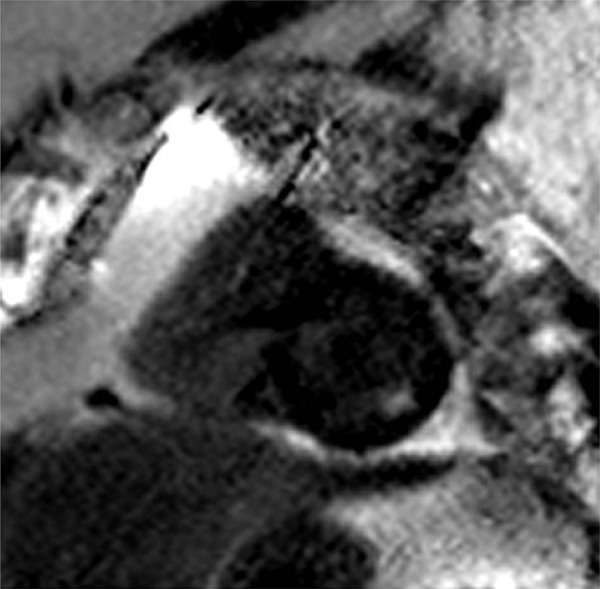
– *Imagem de RMC com sequência PSIR pela técnica do contraste tardio no eixo curto, revelando áreas de realce focal nas regiões subepicárdica e subendocárdica localizadas nas paredes inferior e inferolateral do VE.*

**Figura 9:**
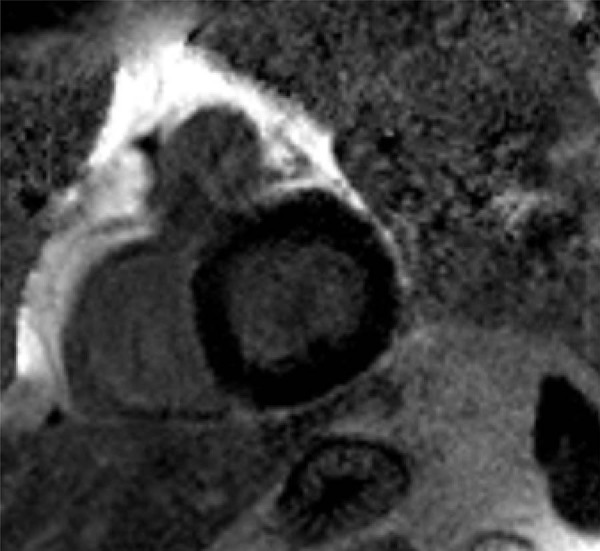
– *Essa imagem mostra a aparência de um miocárdio normal. Essa imagem em particular foi adquirida na sequência PSIR. Não há realce anormal.*

As características demográficas e os graus de fluxo TIMI não foram diferentes no grupo positivo à RCM quando comparado com o grupo negativo à RMI. ( Tabela 2 ). Os níveis de NT-proBNP foram estatisticamente significativos nos pacientes com fluxo lento e tecido cicatricial à RMC (p=0,022) ( Tabela 2 ).

**Tabela 2:** – Características clínicas dos pacientes com fluxo lento

Parâmetros	Fluxo Lento (N= 19)		

	RMI cardíaca (+) (N=10)	RMI cardíaca (-) (N= 9)	Valor de p
Idade, média (DP), anos	54,1 ± 9,6	49,4 ± 7,1	0,29
Sexo (Masculino), n (%)	6 (60)	9 (100)	0,08
Hipertensão, n (%)	4 (40)	2 (22,2)	0,62
Diabetes mellitus, n (%)	3 (30)	3 (33,3)	1,0
Tabagista, n (%)	6 (60)	3 (33,3)	0,37
Histórico familiar, n (%)	1 (10)	3 (33,3)	0,30
Dislipidemia, n (%)	3 (30)	0 (0)	0,21
IMC, média (DP) (kg/m ^2^ )	28,2 ± 3,0	28,0 ± 2,4	0,81
NT-proBNP (pg/ml)	147,10	28,0 (21,5-56,2)	0,03
cTFC (quadro/segundo)	(41,57-734,57)	26,4 (22,9-35,0)	0,67
METs, mL/kg/dk	24,1 (23,8-28,9)	10,26 ± 1,92	0,304

*IMC: índice de massa corporal; cTFC: TIMI corrigido; METs: equivalentes metabólicos; RMI: ressonância magnética por imagem.*

Todos os indivíduos completaram o teste ergométrico usando o protocolo Bruce. Todos os pacientes apresentaram capacidade aeróbica acima de 7 METs. Os testes de esforço foram encerrados a pedido dos próprios pacientes. Não foram observadas alterações significativas de segmento ST (≥1 mm) ou de onda T em nenhum teste de esforço. Os valores do equivalente metabólico (METS) foram diferentes nos grupos controle, fluxo lento, e positivo à RMI (11,15 ± 1,43; 9,74 ± 2,05; 9,27 ± 2,15, respectivamente; p=0,027 para o grupo controle versus o grupo fluxo lento; p=0,013 para o grupo controle versus o grupo positivo à RM). Não houve diferenças de valores de MET entre o grupo controle e o grupo negativo à RM (11,15 ± 1,43 vs 9,74 ± 2,05; p=0,201) ( Tabela 3 ).

**Tabela 3:** – Resultados dos testes de esforço por grupos

Parâmetro	Controle (N= 16) (1)	RM Cardíaca (+) (N=10) (2)	RM Cardíaca (-) (N= 9) (3)	Valor de p (1-2)	Valor de p (1-3)
METs, mL/kg/dk	11,15 ± 1,43	9,27 ± 2,15	10,26 ± 1,92	0,013	0,201

*METs: equivalentes metabólicos; RM: ressonância magnética*

## Discussão

O principal achado deste estudo foi a detecção de tecido cicatricial à RMC em pacientes com fluxo lento na DAE. Os valores de NT-proBNP foram mais altos nos pacientes com fluxo lento e tecido cicatricial à RMC. Além disso, a capacidade aeróbica desses pacientes foi inferior quando comparada com a do grupo controle. Na literatura, os pacientes com fluxo coronário lento não haviam sido previamente avaliados por meio de RMC para detecção de presença de fibrose miocárdica. No nosso estudo, detectamos tecido cicatricial miocárdico na região da DAE em aproximadamente metade dos pacientes com o fenômeno do FCL na DAE. Esse achado foi estatística e clinicamente significativo quando comparado com o grupo controle. Entretanto, nós não avaliamos os pacientes com exames seriados de RMI ou medições seriadas de NT-proBNP. A ausência de tecido cicatricial nos pacientes restantes pode ser explicada pela limitação do nosso estudo. O desenvolvimento de tecido cicatricial no miocárdio requer a existência de um processo progressivo decorrente de danos contínuos durante anos. Assim sendo, a ausência de tecido cicatricial poderia ser o resultado do momento da avaliação. Como bem se sabe, o processo de formação de placa ateromatosa demora muitos anos, dependendo da presença de fatores de risco cardiovasculares, condições ambientais, fatores genéticos, e período de tempo. Os mesmos fatores podem também se aplicar ao fluxo coronário lento. Demonstramos com este estudo que o FLC não é de forma alguma inofensivo e que ele pode levar à cicatrização do tecido miocárdico ao final do respectivo processo patológico. O papel do NT-proBNP na fisiopatologia do FCL não está claro. Demonstrou-se que o peptídeo natriurético do tipo B é secretado dos cardiomiócitos em resposta à isquemia, e que essa secreção pode também ser independente do estresse da parede ventricular esquerda. ^17 - 20^ Adicionalmente, além dos miócitos cardíacos, os fibroblastos podem secretar BNP e causar fibrose por indução das metaloproteinases de matriz através da liberação de BNP. ^21^ No nosso estudo, os níveis de NT-proBNP não foram significativamente altos nos pacientes com fluxo lento. Entretanto, eles foram considerados altos nos pacientes com fluxo lento, nos quais as cicatrizes foram detectadas na RMC. Pode-se sugerir que os níveis de NT-proBNP foram elevados apenas na presença de fibrose suficiente em resposta ao fluxo coronário lento, que levou ao desenvolvimento de tecido cicatricial do miocárdio. A etiologia do FCL não foi claramente compreendida desde que foi descrita pela primeira vez. Embora o FCL possa ser uma consequência de alterações microvasculares, o aumento da resistência microvascular e a aterosclerose generalizada em estágio inicial também demonstraram desempenhar um papel na etiologia. ^22 , 23^ Além disso, tentou-se lançar mão das alterações histológicas e patológicas nas artérias coronárias para elucidar a etiologia. Em um estudo conduzido em pacientes com FCL, Mangieri et al., ^17^ encontraram mudanças, tais como edema celular, hiperplasia fibromuscular, hipertrofia medial, proliferação miointimal, fibrose irregular, lesão capilar e redução do lúmen capilar, resultantes da biópsia do miocárdio, tendo alegado que essas alterações patológicas tornavam o fluxo sanguíneo mais lento através do aumento da resistência vascular. ^22 , 23^ Ademais, no FCL, a ultrassonografia intravascular (USIV) revelou espessamento intimal difuso e calcificação generalizada, e a angigrafia coronária mostrou placas ateromatosas que não causam irregularidade luminal. ^24^

Embora tenha-se relatada associação do fluxo coronário lento com muitas condições patológicas, ele parece desencadear uma doença aterosclerótica generalizada que é coincidente com uma doença microvascular na qual a disfunção endotelial encontra-se em primeiro plano. Pode-se considerer que a fibrose e a isquemia microvascular se desenvolvem no tecido miocárdico de pacientes com FCL, como consequência das alterações que ocorrem no nível microvascular, e nosso estudo corrobora esse ponto de vista.

A função microvascular coronária deteriorada no FCL demonstrou uma relação com o aumento do risco de eventos cardiovasculares. ^25 - 27^ Além disso, foi relatado que, nos pacientes com disfunção microvascular, o prognóstico é semelhante àquele observado na doença arterial coronariana obstrutiva, e que essa disfunção não é tão benigna quanto se pensava. ^28 - 30^ As manifestações clínicas dessa patologia também estão associadas a achados significativos. Dor torácia atípica, ^16 , 31^ dor torácia típica ^32^ e dor torácica em repouso, que requerem intervenção urgente, ^4 , 33^ frequentemente ocorrem em pacientes com fluxo coronário lento. Do mesmo modo, pacientes com FCL mostraram ser mais sintomáticos e suas internações hospitalares mais frequentes. ^34^ Com base nisso, a RMC pode ser considerada uma boa escolha para investigar se o tecido miocárdico foi ou não afetado, além de oferecer uma opção favorável para avaliar a extensão da lesão, nos pacientes com FCL. A RMC de realce tardio possui alta resolução espacial. Com esse método, a fronteira entre o tecido infartado na parede do VE e o miocárdio viavél pode ser identificada através do exame da área do fluxo coronário lento. Além disso, a expansão transmural da área infartada pode ser determinada através desse método. Também é possível distinguir entre a isquemia vascular e não vascular em função da difusão do gadolínio.8 Na cardiomiopatia não isquêmica, o envolvimento do gadolínio é independente da perfusão vascular e ocorre na região subendocárdica. O envolvimento do gadolínio está diretamente associada à alimentação vascular na cardiomiopatia isquêmica. Além disso, esse envolvimento está localizado na região subendocárdica ou transmural. ^35^

Panting et al.,31 demonstraram a hipoperfusão subendocárdica usando a RMC em pacientes com síndrome X, que acredita-se estar associada à disfunção microvascular. ^36^ Do mesmo modo, Lanza et al.,32 detectaram defeitos de perfusão na região da DAE do miocárdio nos pacientes com síndrome X. ^37^ Demonstrou-se também que há uma importante relação entre a reserva de perfusão miocárdica, que é examinada através da RMC, e a disfunção microvascular coronariana, a qual é um precursor da aterosclerose precoce . ^38^

O NT-proBNP deve ser considerado após teste de esforço em pacientes com fluxo coronário lento. Ele pode fornecer informações sobre a fibrose cardíaca, embora ela possa ser afetada por diversas condições. Entretanto, não é possível realizar a RMC em todos os pacientes com contagem baixa de quadros TIMI devido à relação custo-benefício. A RMC pode ser considerada nos casos de pacientes com fluxo coronário lento em grau severo, dor torácica severa e valores elevados do biomarcador após o exercício. Devido ao pequeno número de pacientes do nosso estudo, não podemos fazer nenhuma recomendação em termos de tratamento, RMC ou controle do biomarcador. Todavia, este estudo poderá lançar luz sobre outros estudos, tanto no tocante ao tratamento (drogas antifibróticas) quanto aos exames (RMC, nível de NT-ProBNP, dentre outros).

### Limitações do Estudo

Nosso estudo teve algumas limitações. Primeiramente, o número de pacientes foi pequeno. Em segundo lugar, as angiografias coronarianas foram realizadas por médicos diferentes e, embora as imagens angiográficas sejam padronizadas, houve diferenças pouco significativas entre as projeções. Finalmente, a técnica de ultrassom intravascular (USIV), capaz de mostrar a estrutura e as funções das artérias coronárias em detalhes, as medições da reserva de fluxo fracionada (RFF) e da pressão intracoronária ( *pressure wire* ), e os testes de acetilcolina não foram realizados no nosso estudo. Entretanto, a realização desses testes invasivos, com o seu potential de complicações, em pacientes sem estenose epicárdica não é apropriado por razões éticas.

## Conclusão

Neste estudo, que foi conduzido para demonstrar o tecido cicatricial relacionado com o fenômeno do FLC, a RMC pela técnica do realce tardio apresentou resultados positivos. A RMC revelou tecido cicatricial nos pacientes com fluxo lento. Esses resultados sugerem que o fenômeno do fluxo lento pode acarretar alterações irreversíveis no tecido do miocárdio. As prováveis consequências dessas alterações devem ser investigadas em pesquisas futuras.
