# Supplementary material for: Determinação do Tecido Cicatricial do Miocárdio no Fenômeno de Fluxo Coronário Lento e a Relação entre a Quantidade de Tecido Cicatricial e o Nt-ProBNP
Source: Arq Bras Cardiol. 2020 Mar 20;114(3):540–51. [Article in Portuguese] doi: 10.36660/abc.2018149 (PMC7792723; doi:10.36660/abc.2018149)
Supplement: Translation

# Translation: Determination of Myocardial Scar Tissue in Coronary Slow Flow Phenomenon and The Relationship Between Amount of Scar Tissue and Nt-ProBNP

**Authors:** Mustafa Candemir, Asife Şahinarslan, Merve Yazol, Yusuf Ali Öner, Bülent Boyacı

**Affiliations:** 1 Turkey Yozgat City Hospital - Department of Cardiology, Yozgat - Turkey; 2 Turkey Gazi University - Faculty of Medicine - Department of Cardiology, Ankara - Turkey; 3 Turkey Şanlıurfa Education and Research Hospital, Department of Radiology, Şanlıurfa - Turkey; 4 Turkey Gazi University - Faculty of Medicine - Department of Radiology, Ankara - Turkey

**Keywords:** Heart Failure, Fractional Flow Reserve, Myocardial, Cicatrix, Hypertrophic, Prognosis, Natriuretic-Peptide, C-Type, Endomyocardial Fibrosis, Magnetic Resonance Spectroscopy

## Abstract

**Background:**

Pathophysiology and prognosis are not clearly determined in patients with the coronary slow flow phenomenon (CSFP). These patients present with various clinical conditions ranging from being asymptomatic to being admitted with sudden cardiac death.

**Objectives:**

We aimed at assessing the findings of late gadolinium enhancement (LGE) in cardiac magnetic resonance imaging (CMR) as an indicator of myocardial fibrosis. We also aimed at determining the relationship between the presence of myocardial fibrosis and NT-proBNP levels in patients with CSFP in the left anterior descending coronary artery (LAD).

**Methods:**

A total of 35 patients were enrolled within an age range of 31-75. The study patients (n=19) had normal epicardial coronary arteries at angiography, but they presented with CSFP in the LAD. The control group patients (n=16) had normal epicardial coronary arteries and TIMI scores at normal levels in angiography. In both groups, the patients were examined with CMR for the presence of myocardial fibrosis. In addition, plasma NT-proBNP levels were measured. A p-value < 0.05 was considered significant.

**Results:**

The rate of myocardial fibrosis was significantly higher in CMR in the patients with CSFP (p=0.018). A variable amount of myocardial scar tissue was detected at the left ventricular apex in 7 patients and at the inferior and inferolateral regions in 3 patients. There was no difference in the level of NT-proBNP in patients with CSFP. However, the NT-proBNP levels were higher in patients with CSFP, who had scar tissue in CMR (p=0.022).

**Conclusions:**

In conclusion, LGE in CMR showed that ischemic myocardial scarring may exist in patients with CSFP. These results indicate that CSFP may not always be innocent. (Arq Bras Cardiol. 2020; 114(3):540-551)

## Introduction

There is limited information in the literature regarding the prognosis of slow coronary flow phenomenon (CSFP). The preexisting data indicates that slow flow-related myocardial ischemia may cause angina and the prognosis is worse in these patients. ^1^ Acute myocardial infarction, ^2^ sudden cardiac death and malignant ventricular arrhythmia were also reported to be associated with CSFP. ^3^ The occurrence of recurrent episodes of chest pain or chest pain developing at rest, as well as high rates of emergency admissions, and hospitalizations are reported. ^4 , 5^ Thus, this phenomenon is not as innocent as it appears to be, bearing a potential to cause serious deterioration in the quality of life. It is not clearly known today whether organic injuries exist in these patients, due to the lack of further investigation and findings.

The level of N-terminal proB-type natriuretic peptide (NT-proBNP) has been shown to increase after exercise in patients with slow coronary artery flow. ^6^ There is a correlation between ischemia or infarct size on magnetic resonance imaging (MRI) and this correlation can be observed in the NT-proBNP levels in patients with acute coronary syndrome as well. ^7^ As a result of the advances in cardiac magnetic resonance imaging (CMR), microvascular ischemia and cardiac fibrosis can be demonstrated using this technique. ^8 , 9^ The relation between the extent of fibrosis and NT-proBNP levels in these patients has been revealed by several MRI studies performed in patients with acute coronary syndrome. ^10 , 11^ However, there are no studies in the literature evaluating patients with CSFP for the presence of fibrosis in the myocardial tissue according to the findings of late gadolinium enhancement in CMR. This study aimed at investigating the presence of myocardial fibrosis in patients with slow flow in the left anterior descending coronary artery by using the late gadolinium enhancement technique in CMR. In addition, it aimed at evaluating the relationship between myocardial fibrosis and NT-proBNP levels.

## Materials and Methods

### Study Population

Among the patients who were admitted to our department between January 2015 - August 2016 and who underwent coronary angiography for chest pain, 19 patients with the coronary slow flow phenomenon in LAD were included in this prospective cohort study. The control group included sixteen patients whose epicardial arteries were entirely normal with a normal coronary flow.

This study was approved by the ethics committee of the Gazi University Hospital and was conducted in accordance with the principles of the Helsinki declaration.

### Exclusion Criteria

The following patients were excluded from the study: patients with coronary artery ectasia or atherosclerotic lesions in left main and left anterior descending coronary arteries; patients who underwent percutaneous coronary intervention; patients scheduled to undergo coronary artery intervention; patients with >50% stenosis in any coronary artery; patients with a prior history of MI; patients with <50% left ventricular systolic function; patients with claustrophobia, heart failure or valve dysfunction, ventricular extrasystoles or atrioventricular conduction abnormalities, and branch block or atrial fibrillation; patients with positive treadmill test; patients with restrictive, hypertrophic or dilated cardiomyopathies; patients with known systemic disease (hyperthyroidism, hypothyroidism, malignancy, autoimmune disease, infection or any of the pulmonary, hepatic, renal, hematologic disorders); patients with a history of myocarditis, whose GFRs are <80 ml / min and patients who refused to participate in the study.

### Patient Data

The study patients were measured for height, weight, and body mass index (BMI). Patients’ age, gender, cardiovascular risk factors (hypertension, diabetes, dyslipidemia, smoking and family history), demographic characteristics and comorbid diseases were recorded. Electrocardiography (ECG) was obtained for all patients and all of them demonstrated a sinus rhythm. All study patients were examined on the right lateral decubitus position with a Vivid 7-Pro Ultrasound system (Vingmed Electronic, GE, Horten, Norway), equipped with a 2.5 MHz probe, through simultaneous one-lead ECG recording. M-mode and Doppler measurements were performed in accordance with the recommendations of the American Echocardiography Association. ^12^

Exercise test was performed at an average of 3 days before angiography in all patients (GE medical system, Milwaukee, USA), according to the standard Bruce protocol test, with standard ECG, blood pressure and heart rate measurements performed at prespecified time points, as per relevant guidelines. ^13^

Blood samples for the quantification of NT-proBNP were collected through the angiography sheath immediately before its removal. After the collection, they were centrifuged for 10 min at 4500 rpm and stored at -20 ºC until the time when the isolated serum was analyzed. On the day of the analysis, after the samples reached room temperature, an electrochemiluminescence immunoassay was performed with the Roche Cobas e 411 analyzer (Roche Diagnostics GmbH, Mannheim, Germany). The results were presented in picograms per ml (pg/ml). The coefficent of variation value for the NT-proBNP was found below 5% via this method.

### Coronary Angiography and Timi Frame Count

Coronary angiography was performed using the standard Judkins technique with a femoral approach and at 30 frames per second, using Toshiba Infinix cardiac angiography (Toshiba Corporation, Tochigi, Japan). Iopromide (Ultravist-370; Bayer Pharma AG, Berlin, Germany) was used as contrast agent during coronary angiography. An average of 6 to 8 ml of contrast agent was injected manually for each exposure. Coronary arteries were visualized through left and right oblique views with appropiate cranial or caudal angles. The speed of flow at LAD was assessed in right or left anterior oblique views often with caudal angle. The images were evaluated by two clinical specialists who were blind to the clinical findings of the patients.

Quantitative evaluation of coronary flow was performed in accordance with the TIMI-4 study, by counting cine frames, starting from the time of contrast agent administration, until it reached to a certain distal point. ^14^ The methodology of frame count was standardized for each epicardial vessel. TIMI frame counting started with the first frame in which the dye completely filled in the artery. The complete filling of the artery was determined by meeting the following three criteria: (1) A column of almost or fully concentrated dye should extend across the entire width of the origin of the artery; (2) The dye should touch both borders of the origin of the artery; and (3) there should be an antegrade motion of the dye. The last frame counted was the one in which the dye first enterered the end-point branch of the target artery. A complete opacification was not required at the distal segment.

LAD and TIMI frame counts were 1.7 times longer than the mean of the RCA and CX counts. Therefore, the longer LAD frame counts were corrected by dividing by 1.7 to derive the corrected TIMI frame count (CTFC).

In our study, coronary flow for LAD was accepted to be normal when the TIMI frame count was <23 and it was accepted as slow when the TIMI frame count was ≥ 23. ^15 , 16^

### Magnetic Resonance Imaging Technique

CMR was performed after a median of 8 days (range 0-21 days) after coronary angiography. Standard sequences of cardiac MR perfusion studies were used in all patients. The left antecubital vein was used for intravenous contrast injection. MRI scans of patients were obtained using a 3 Tesla MRI device (Siemens MAGNETOM ^®^ Verio, Erlangen, Germany) with a gradient power of 45 mT/m. A 6-channel body coil was placed on the front chest wall while the patient was lying in the supine position with ECG pads placed properly. Multiplanar scout images were obtained with the phase-sensitive inversion-recovery (PSIR) turbo FLASH sequence using a repeated breath-hold MRI-technique. Standart long-axis, 2-chamber, 4-chamber and short- axis images of heart were obtained by aligning the mitral valve and the apex. Imaging parameters were: repetition time (TR) = 800ms; echo time (TE) = 6.66ms; slice thickness = 8 mm; matrix = 128x256 and field of view (FOV) = 400 mm. T1, T2 weighted images accompanied by ‘inversion recovery’ pulse for the suppression of blood signals (dark blood) and the turbo spin echo sequence were obtained in order to evaluate the myocardial morphology (TR/TE/ thickness/ matrix/ FOV: 698/6.6/ 8 mm/ 128x256, 360 mm).

Dynamic first-pass myocardial perfusion imaging with SR Turbo FLASH (TfI) pulse sequence was acquired after 0.025 mmol/kg Gd-DTPA (Magnevist; Bayer Healthcare, Wayne NJ, USA) was administered intravenously. In eight-minute resting intervals, cine short axis gradient echo sequences for functional imaging of the ventricles were obtained throughout cardiac cycle using the breath-hold technique (TR/TE/ thickness/ matrix/ FOV: 40,24/ TE/ 8 mm/128x256/ 360 mm). Short-axis and 4-chamber images were obtained with T1-weighted PSIR technique, approximately in the 8th minute after the contrast was applied (TR/TE/ thickness/ matrix/ FOV: 756/ TE: 1,15/ 6 mm/ 128x256/360 mm). Total imaging duration lasted 35 minutes on average.

The cost of CMR and plasma NT-proBNP testing were covered by the Scientific Research Projects Unit of the Gazi University.

### Magnetic Resonance Image Analysis

All CMR images were transferred to the work station for analysis (Siemens multimodality workplace, Leonardo, Siemens Healthcare). All evaluations were performed visually. CMR studies were retrospectively evaluated by a radiologist experienced for more than 15 years in cardiac imaging, who was blind to the results of echocardiography and coronary angiography examinations. When any perfusion defects or late enhancement were observed during these examinations, they were precisely recorded. Contrast enhancement in perfusion sequences was defined as accomplishing all of the 5 phases after obtaining the highest signal intensity in the left ventricle. CMR results were then compared with the patients’ echocardiography and coronary angiography results.

### Statistical Analysis

The study data were analyzed by using the SPSS (SPSS Inc., Chicago, version 21.0) program. The variables were examined using visual (histograms, probability plots) and analytical methods (the Kolmogorov-Simirnov test) to determine whether or not they were normally distributed. Descriptive analyses were presented with the values and standard deviations for the normally distributed variables, and with the median (interquartile range), for the non-normally distributed variables. Categorical variables were presented using percentages. Independent samples t-test and the Mann-Whitney U test were used to compare the numerical variables. Pearson’s Chi-square analysis was used to compare the categorical data, but Fisher’s exact test was performed when two of the expected values were below 5 or one of the expected value was below 2. A difference with a p-value <0.05 was considered statistically significant.

## Results

A total of 35 patients were included in the study. Patients were divided into 2 groups as the patient group and the control group. Nineteen patients were identified comprising the group with slow flow in LAD, and 16 patients with normal coronary flow were included in the control group ( Figure 1 ). The patients in the control group were matched for their risk factors to the individuals in the patient group. The mean age of the patients was 50.3 ± 10.7, and 6 out of 35 patients (17%) were females. Eleven patients (31%) were diabetic, 9 patients (25%) were hypertensive, 5 patients (14%) were dyslipidemic, 18 patients (51%) were smokers and 8 patients (22%) had a positive family history ( Table 1 ).

**Figure 1:** – *Patient selection. CMR: cardiac magnetic resonance imaging.*

**Table 1:** – Comparison of Clinical Characteristics between both Groups

Parameters	Total (N=35)	Slow Flow (N= 19)	Control (N= 16)	p value
Age, mean (SD), years	50.3 ± 10.7	51.3 ± 8.2	49.44 ± 12.8	0.62
Sex (Male), n (%)	29 (82)	15 (78.9)	14 (87.5)	0.50
Hypertension, n (%)	9 (25)	6 (31.6)	3 (18.8)	0.38
Diabetes mellitus, n (%)	11 (31)	6 (31.6)	5 (31.3)	0.98
Smoker, n (%)	18 (51)	9 (47.4)	9 (56.3)	0.60
Family history, n (%)	8 (22)	4 (21.1)	4 (25)	0.78
Dyslipidaemia, n (%)	5 (14)	3 (15.8)	2 (12.5)	0.78
BMI, mean (SD) (kg/m ^2^ )	27.7 ± 2.3	28.1 ± 2.5	27.3 ± 2	0.39
NT-proBNP (pg/ml)	29,5 (17.7-66.2)	47.8 (22.6-121.5)	26.0 (10.9-58.1)	0.246
cTIMI flow (frame/second)	34.6 ± 16.2	28.0 ± 8.6	13.1 ± 1.2	<0.001
METs, mL/kg/dk	10.38 ± 1.91	9.74 ± 2.05	11.15 ± 1.43	0.027
Positive results of MRI n (%)	10 (28)	10 (52.6)	0 (0)	0.001

*BMI: body mass index ; cTIMI: corrected Thrombolysis in Myocardial Infarction; METs: metabolic equivalents; MRI: magnetic resonance imaging.*

The main complaint was chest pain in all study patients. ECG of all patients was sinus rhythm. Heart rates were between 64/min - 92/min. The average heart rate was 74/min. In addition, there was no sign of ischemia, hypertrophy or arrhythmia in the ECG. Left ventricular ejection fraction and other echocardiography findings of the patients were normal. In addition, all patients’ treadmill tests were negative. High sensitivity troponin was measured before and after coronary angiography in all patients. All values were below the threshold and there was no increase in troponin values after angiography.

The time interval between the CMR examinations and catheter coronary angiography of the patients was scheduled not to be longer than 21 days.

When patients with slow flow were compared with the control group, no significant differences were found in NT-proBNP values (p=0.247). The positive CMR results were significantly more common in the patients with the slow flow (p=0.001) ( Table 1 ). Scar tissue was found at varying levels in the cardiac apex of 7 patients (Figures 2 and 5) and at the inferior and inferolateral regions in 3 patients (Figures 3,4,6,7 and 8). No scar tissues were found in 9 patients ( Figure 9 ).

**Figure 2:** – *Four-chamber delayed-enhancement Phase Sensitive Inversion Recovery (PSIR) image, showing delayed subendocardial circumferential enhancement in the apical region (arrows) of the left ventricle.*

**Figure 5:** – *There is a focal lesion in four-chamber PSIR prepared gadolinium-enhanced T1-weighted image. Focal lesion localized to the apical region of the left ventricle, showing late subendocardial- myocardial enhancement compatible with fibrosis.*

**Figure 3:** – *Short- axis delayed contrast-enhanced PSIR cardiac MR image, showing focal subendocardial transmural and subepicardial enhancement areas, mostly in the inferior and inferolateral left ventricular walls.*

**Figure 4:** – *Short- axis delayed contrast-enhanced PSIR cardiac MR image, demonstrating focal subendocardial and subepicardial enhancement areas localized into the inferior and inferolateral left ventricular walls (arrows). These hyperintense areas reflect scattered fibrotic scar tissues in the LAD territory.*

**Figure 6:** – *Short-axis delayed contrast-enhanced PSIR image represents sub-endocardial enhancement areas, mostly in the inferorolateral left ventricular wall. Scar tissue spanning in nearly 25-50% of wall thickness.*

**Figure 7:** – *Short- axis delayed contrast-enhanced PSIR cardiac MR image, showing focal subendocardial transmural and subepicardial enhancement areas, mostly in the inferior and inferolateral left ventricular walls (arrows), indicating scar tissues in the distribution of the LAD.*

**Figure 8:** – *Short- axis delayed contrast-enhanced PSIR cardiac MR image, demonstrating focal subendocardial and subepicardial enhancement areas localized into the inferior and inferolateral left ventricular walls.*

**Figure 9:** – *This image shows the appearance of normal myocardium. This particular image was obtained using Phase Sensitive Inversion Recovery (PSIR). There is no abnormal enhancement.*

Demographic characteristics and TIMI grade flow were not different in the CMR positive group compared to the MRI negative group ( Table 2 ). NT-proBNP levels were statistically significant in patients with slow flow and scar tissue in CMR (p=0.022) ( Table 2 ).

**Table 2:** – Clinical characteristics in patients with slow flow

Parameters	Slow Flow (N= 19)		

	Cardiac MRI (+) (N=10)	Cardiac MRI (-) (N= 9)	p value
Age, mean (SD), years	54.1 ± 9.6	49.4 ± 7.1	0.29
Sex (Male), n (%)	6 (60)	9 (100)	0.08
Hypertension, n (%)	4 (40)	2 (22.2)	0.62
Diabetes mellitus, n (%)	3 (30)	3 (33.3)	1.0
Smoker, n (%)	6 (60)	3 (33.3)	0.37
Family history, n (%)	1 (10)	3 (33.3)	0.30
Dyslipidaemia, n (%)	3 (30)	0 (0)	0.21
BMI, mean (SD) (kg/m ^2^ )	28.2 ± 3.0	28.0 ± 2.4	0.81
NT-proBNP (pg/ml)	147.10	28.0 (21.5-56.2)	0.03
cTIMI flow (frame/second)	(41.57-734.57)	26.4 (22.9-35.0)	0.67
METs, mL/kg/dk	24.1 (23.8-28.9)	10.26 ± 1.92	0.304

*BMI: body mass index ; cTIMI: corrected Thrombolysis in Myocardial Infarction; METs: metabolic equivalents; MRI: magnetic resonance imaging.*

All subjects completed treadmill exercise testing using the Bruce protocol. All patients had exercise capacity over 7 mets. Treadmill tests were terminated on the patients’ own request. There was no significant ST depression (≥1 mm) or T negativity in any exercise test. Metabolic equivalent (MET) values were different in the control, slow flow, and MR positive groups (11.15 ± 1.43; 9.74 ± 2.05; 9.27 ± 2.15, respectively; p=0.027 for the control group vs. the slow flow group; p=0.013 for the group control vs. the MR positive group). There were no differences in MET values between the control group and the MR negative group (11.15 ± 1.43 vs 9.74 ± 2.05; p=0.201) ( Table 3 ).

**Table 3:** – Exercise test results for groups

Parameter	Control (N= 16) (1)	Cardiac MRI (+) (N=10) (2)	Cardiac MRI (-) (N= 9) (3)	p value (1-2)	p value (1-3)
**METs, mL/kg/dk**	11.15 ± 1.43	9.27 ± 2.15	10.26 ± 1.92	0.013	0.201

*METs: metabolic equivalents; MRI: magnetic resonance imaging.*

## Discussion

The main finding of this study was the detection of scar tissue in CMR in the patients with slow flow in the LAD. NT-proBNP values were higher in patients with slow flow and scar tissue in CMR. In addition, the exercise capacity of these patients was lower compared to the control group. Coronary slow flow patients have not previously been evaluated with CMR to detect any presence of myocardial fibrosis in the literature. In our study, we detected scar tissue in the myocardium in the LAD field in about half of patients with CSFP in the LAD. This finding was statistically and clinically significant when compared to the control group. However, we did not evaluate the patients with serial MRI examinations or serial NT-proBNP measurements. The absence of scar tissue in the remaining patients may be explained by this limitation of our study. The development of scar tissue in the myocardium requires a progressive process occurring as a result of continuous damage over years. Thus, the absence of scar tissue might have been due to timing of the assessment. As it is very well known, the process of atheromatous plaque formation takes many years, depending on the presence of cardiovascular risk factors, environmental conditions, genetic factors and the time period. The same factors may also apply to coronary slow flow. We have shown with this study that the CSFP is not harmless at all and that it can lead to scarring in the myocardial tissue at the end of the respective pathological process.

The role of NT-proBNP in the pathophysiology of CSFP is not clear. It has been shown that B-type natriuretic peptide is secreted from cardiomyocytes in response to ischemia and that its secretion can also be independent of left ventricular wall stress. ^17 - 20^ Also, in addition to cardiac myocytes, fibroblasts can secrete BNP and cause fibrosis by induction of matrix metalloproteinases by releasing BNP. ^21^ In our study, the levels of NT-proBNP were not significantly high in patients with slow flow. However, they were found to be high in patients with slow flow, in whom scars were detected in CMR. It may be suggested that NT-proBNP levels are elevated only in the presence of sufficient fibrosis in response to coronary slow flow, which has led to the development of myocardial scar tissue. The etiology of CSFP has not been clearly understood since it was first described. Although CSFP may be the result of microvascular alterations, increased microvascular resistance and early stage widespread atherosclerosis have also been shown to play a role in the etiology. ^22 , 23^ In addition, histological and pathological changes in the coronary arteries have been tried to be used for elucidating the etiology. In a study conducted in patients with CSFP, Mangieri et al. ^17^ found changes such as cellular edema, fibromuscular hyperplasia, medial hypertrophy, myointimal proliferation, irregular fibrosis, capillary damage and decreased capillary lumen, as a result of myocardial biopsy, and claimed that these pathological changes slowed blood flow by increasing vascular resistance. ^22 , 23^ Moreover, in CSFP, intravascular ultrasonography (IVUS) has shown diffuse intimal thickening and widespread calcification, and coronary angiography has shown atheromatous plaques that do not cause luminal irregularity. ^24^

Although coronary slow flow has been reported to be associated with many pathologic conditions, it appears to be the onset of a widespread atherosclerotic disease that is coincidental with a microvascular disease in which endothelial dysfunction is in the forefront. It can be considered that microvascular ischemia and fibrosis may develop in the myocardial tissue in patients with CSFP as a result of changes that take place at the microvascular level and our study supports this view.

The deteriorated coronary microvascular function in CSFP has been shown to be associated with increased risk of cardiovascular events. ^25 - 27^ It has also been reported that, in patients with microvascular dysfunction, the prognosis is similar to that observed in obstructive coronary artery disease, and that this dysfunction is not as benign as it is thought to be. ^28 - 30^ Clinical manifestations of this pathology are also associated with significant findings. Atypical chest pain, ^16 - 31^ typical chest pain ^32^ and resting chest pain that require urgent intervention ^4 , 33^ frequently occur in patients with coronary slow flow. Similarly, patients with CSFP were found to be more symptomatic and their hospital admissions were found to be more frequent. ^34^ Based on this, CMR may be considered a good choice for investigating whether the myocardial tissue is affected or not, as well as providing a favorable option to evaluate the extent of the injury in patients with CSFP. Delayed contrast-enhanced CMR has high spatial resolution. With this method, the boundary between the infarcted tissue on the LV wall and the viable myocardium can be identified by examining the area of coronary slow flow. In addition, the transmural spread of the infarction area can be determined with this method. It is also possible to distinguish between vascular and non-vascular ischemia owing to the diffusion of gadolinium. ^8^ In non-ischemic cardiomyopathy, gadolinium involvement is independent of vascular perfusion and occurs in the subendocardial region. Gadolinium involvement is directly associated with vascular feeding in ischemic cardiomyopathy. In addition, this involvement is in the subendocardial or transmural region. ^35^

Panting et al. ^31^ demonstrated subendocardial hypoperfusion with CMR in patients with syndrome X, which is believed to be associated with microvascular dysfunction. ^36^ In the same way, Lanza et al. ^32^ detected perfusion defects in the LAD region of the myocardium in syndrome X patients. ^37^ It is also shown that there is an important relationship between a myocardial perfusion reserve, which is examined with CMR and coronary microvascular dysfunction, and is a precursor of early atherosclerosis. ^38^

NT-proBNP may be considered after an effort test in patients with coronary slow flow. It can give information about cardiac fibrosis, although it may be affected by several conditions. However, it is not possible to perform a CMR in all patients with low TIMI frame count due to cost effectiveness. CMR may be considered in patients with severe coronary slow flow degree, severe chest pain and high biomarker values after exercise. Because of the small number of patients in our study, we cannot make any recommendations about treatment, CMR or biomarker control. However, this study will shed light on studies on both treatment (anti-fibrotic drugs) and examination (CMR, NT-ProBNP, among others).

### Study Limitations

Our study had a few limitations. First, the number of patients was low. Second, coronary angiographies were performed by different clinicians and, although angiographic images were standardized, there were negligible differences between the projections. Finally, the intravascular ultrasound (IVUS) technique, which can show the structure and functions of coronary arteries in detail, fractional flow reserve (FFR) and intracoronary pressure (pressure-wire) measurements, and acetylcholine testing were not performed in our study. However, performing these invasive tests, with their potential complications, in patients with no epicardial stenosis is not appropriate due to ethical reasons.

## Conclusion

In this study, which was conducted to demonstrate scar tissue related to CSFP, CMR with delayed gadolinium enhancement technique has been found to yield valuable results. CMR showed scar tissue in patients with slow flow. These results suggest that the slow flow phenomenon may result in irreversible changes in myocardial tissue. The probable consequences of these changes should be investigated in further studies.
